# Supplementary material for: Connectomic and behavioural alterations in creatine transporter deficiency are partially normalized by gene therapy
Source: Brain. 2025 Jul 29;149(3):1043–57. doi: 10.1093/brain/awaf275 (PMC13016649; doi:10.1093/brain/awaf275)
Supplement: awaf275_Supplementary_Data [file awaf275_supplementary_data.zip › brain-2024-01184-File010.pdf]

Supplementary figures and tables

Connectomic and behavioral alterations in creatine transporter deficiency are partially normalized by gene therapy

Caterina Montani[1*§], Ludovica Iovino[2§], Federica Di Vetta[2,3§], Jean-Charles Rene' Pasquin Mariani[1], A. Elizabeth De Guzman[1], Silvia Gini [1], Alberto Galbusera[1], Bianca D'Epifanio[1], Elsa Ghirardini[2,4], Sara Cornuti[5], Lorenzo Dadà[5,6], Elena Putignano[2], Maria Grazia Alessandrì[4], Giulia Vasirani[7,8], Sine Mandrup Bertozzi[9], Andrea Armirotti[9], Laura Baroncelli[2,4+] and Alessandro Gozzi[1+]

Author affiliations:

[1]Functional Neuroimaging Laboratory, Istituto Italiano di Tecnologia, Center for Neuroscience and Cognitive Systems, CNCS@UNITN, Rovereto, Italy

[2] Institute of Neuroscience, National Research Council (CNR), Pisa, Italy

[3] Department of Biology, University of Pisa, Pisa, Italy

[4] Department of Developmental Neuroscience, IRCCS Stella Maris Foundation, Pisa, Italy

[5]BIO@SNS lab, Scuola Normale Superiore di Pisa, Pisa, Italy

[6] Department of Statistics, Computer Science, Applications "Giuseppe Parenti" (DiSIA), University of Florence, Florence, Italy

[7]University of Genova, Genova, Italy

[8] IRCCS Ospedale Policlinico San Martino, Genova, Italy

[9]Analytical Chemistry Facility, Istituto Italiano di Tecnologia, Genova, Italy

[*] Current address: IRCCS Ospedale Policlinico San Martino, Genova, Italy

[§] Contributed equally

[+] Contributed equally

Correspondence to:
Alessandro Gozzi
Functional Neuroimaging Laboratory,
Istituto Italiano di Tecnologia,
Center for Neuroscience and Cognitive Systems, CNCS@UNITN,
Rovereto, Italy
alessandro.gozzi@iit.it

Caterina Montani
IRCCS Ospedale Policlinico San Martino,
Genova, Italy
caterina.montani@hsanmartino.it

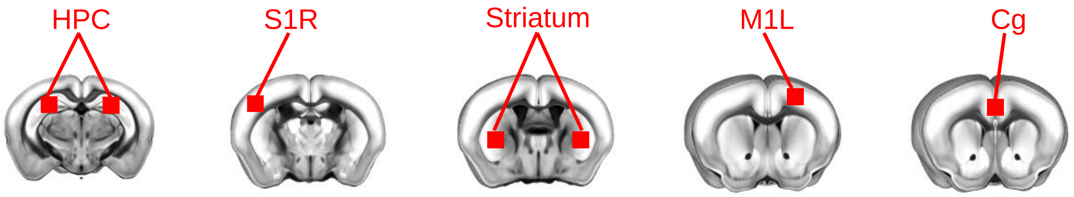

Supplementary Figure 1. Anatomical location of seed regions. *HPC, hippocampus; S1R, right somatosensory cortex 1; M1L, left motor cortex; Cg, cingulate cortex.*

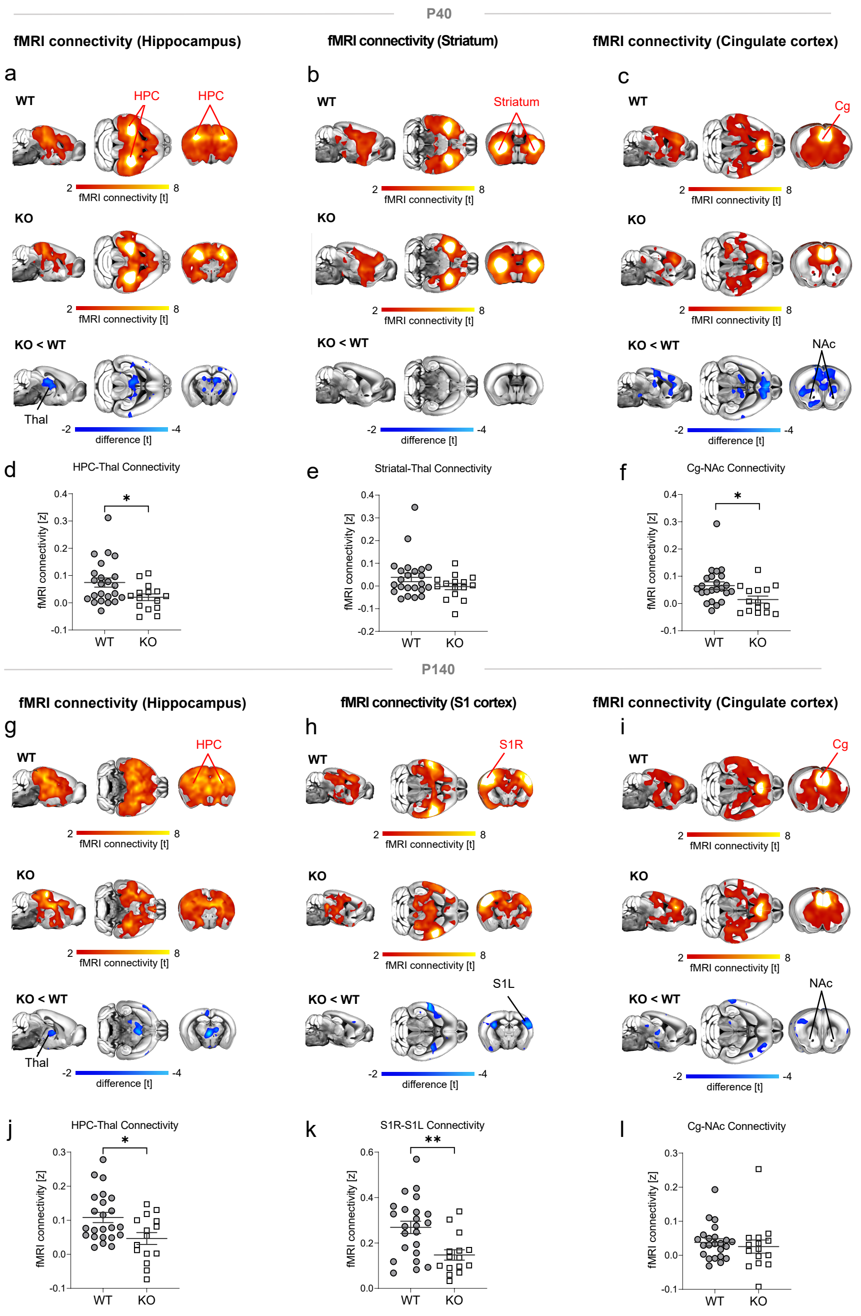

Supplementary Figure 2. Seed-based analysis of fMRI connectivity. *(a-f) Seed-correlation mapping highlighted disrupted hippocampal-thalamus and cingulate cortex connectivity in Slc6a8-KO mice at P40. (g-l) fMRI connectivity in the hippocampal circuit was disrupted also at P140. At this age we also found a prominent reduction of inter-hemispheric somatosensory connectivity. Red/yellow indicates areas in the brain maps exhibiting significant (t> 2.1) fMRI connectivity with seed regions (indicated with red lettering). Blue indicates between-group connectivity differences (t-test, t> 2.1). Corresponding quantifications of connectivity changes in the two groups are reported in panels d-f for P40 (HPC-Thal, t-test, t= 2.35, p= 0.025; Cg-NAc, t-test; t= 2.6, p= 0.014) and j-l for P140 (HPC-Thal, t-test; t= 2.65, p= 0.012; S1R-S1L, t-test; t = 3.17, p = 0.003). HPC, hippocampus; Thal, thalamus; Cg, cingulate cortex; NAc, Nucleus Accumbens; S1R, right somatosensory cortex; S1L, left somatosensory cortex. All statistics are FWE cluster-corrected. FWE, family-wise error. *p < 0.05, **p < 0.01. Error bars indicate SEM and dots represent individual values.*

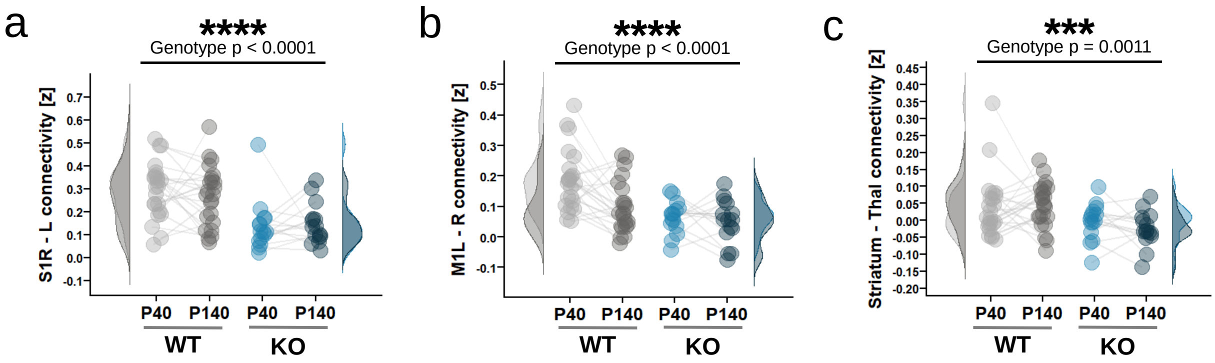

Supplementary Figure 3. Temporal evolution of fMRI connectivity in WT and Slc6a8-KO mice at P40 and P140. *Quantification and distribution of fMRI connectivity in somatosensory (a, two-way RM ANOVA, genotype factor F = 26, p < 0.0001, genotype x age interaction F = 0.43, p = 0.512) and motor cortices (b, genotype factor F = 14.2, p = 0.0003, genotype x age interaction F = 2.8, p = 0.1) and between striatal and thalamic regions (c, genotype factor, F = 11.4, p = 0.0012, interaction F = 0.85, p = 0.36) in WT and Slc6a8-KO mice administered with AAV-GFP, at P40 and P140. ***p < 0.001, ****p<0.0001. L, left; R, right; M1, motor cortex; S1, somatosensory cortex; Thal, thalamus. Dots represent individual values.*

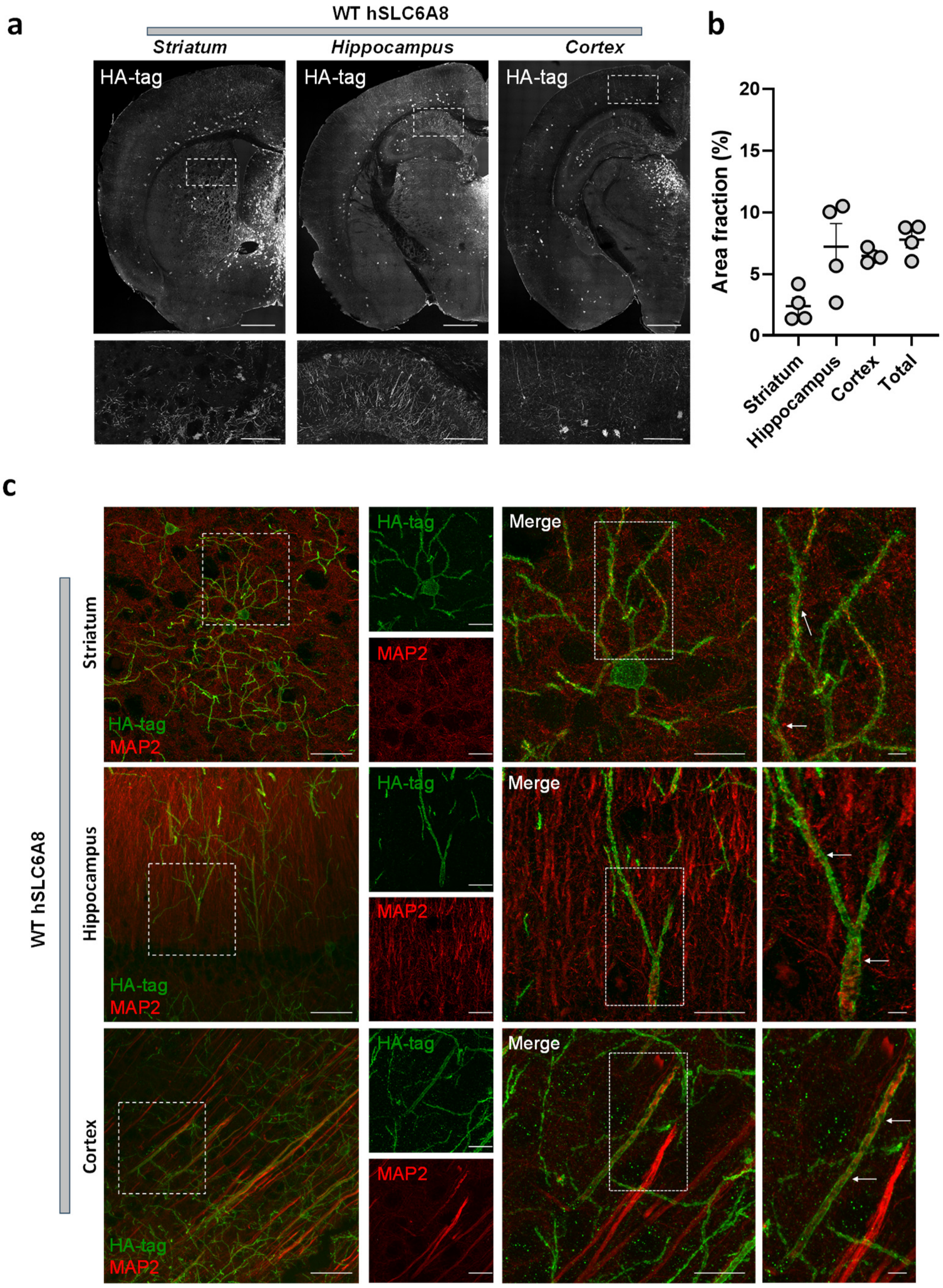

Supplementary Figure 4. Exogenous CRT protein is widely distributed throughout the brain and colocalizes with neurons in different brain regions in WT mice. *(**a**) Representative coronal sections (20x magnification) stained to quantify HA-tagged distribution of transgenic CRT protein in the striatum, hippocampus and cerebral cortex of WT mice treated with AAV-hSLC6A8. Scale bar are 1 mm top panels, 100 µm bottom panels. (**b**) Quantification of the area positive for HA-tag staining (%) in the three brain regions considered and in the right brain hemisphere (total) of WT mice. Error bars indicate SEM. (**c**) Representative images illustrating colocalization of HA-tag staining (green signal) along with the neuronal marker MAP2 (red signal) across different brain regions. Panels on the leftmost column were acquired at 20x magnification (scale bars are 50 µm). Additional panels were acquired at 63x magnification (scale bars are 20 µm, 50 µm and 5 µm from left to right, respectively).*

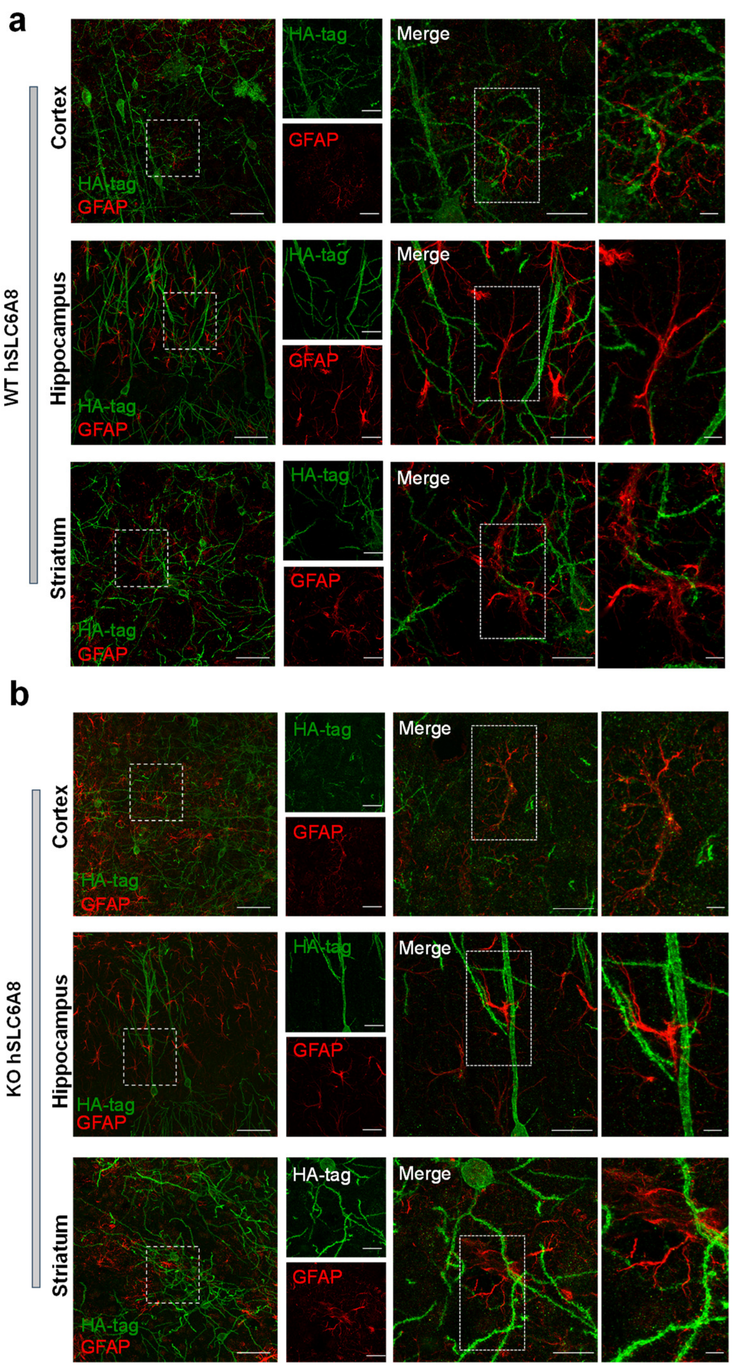

Supplementary Figure 5. Exogenous CRT protein is not expressed in astrocytes. *Representative images illustrating colocalization of HA-tag staining (green signal) with the astrocytic marker GFAP (red signal) in the cortex, hippocampus and striatum of WT* ***(a)*** *and Slc6a8-KO* ***(b)*** *mice treated injected with AAV-hSLC6A8. In both (a) and (b), images in the leftmost columns were acquired at 20x magnification (scale bars are 50 µm). Additional panels were acquired at 63x magnification (scale bars are 20 µm, 50 µm and 5 µm from left to right, respectively).*

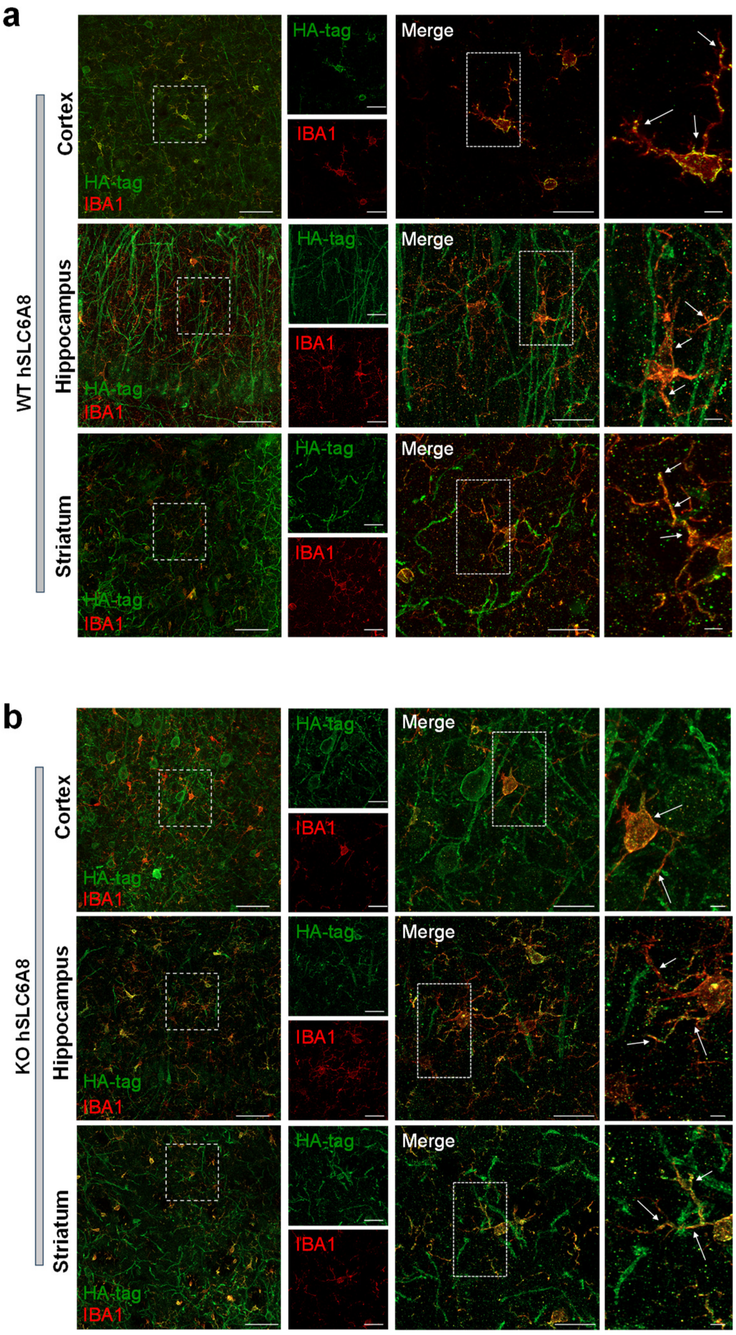

Supplementary Figure 6. Colocalization of exogenous CRT protein with the microglial marker IBA1. *Representative images at 20x and 63x magnification, and relative insets illustrating the colocalization of HA-tag staining (green signal) with the microglial marker IBA1 (red signal) in the cortex, hippocampus and striatum of WT **(a)** and Slc6a8-KO **(b)** mice injected with AAV-hSLC6A8 at P1. In both (a) and (b), images in the leftmost columns were acquired at 20x magnification (scale bars are 50 µm). Additional panels were acquired at 63x magnification (scale bars are 20 µm, 50 µm and 5 µm from left to right, respectively).*

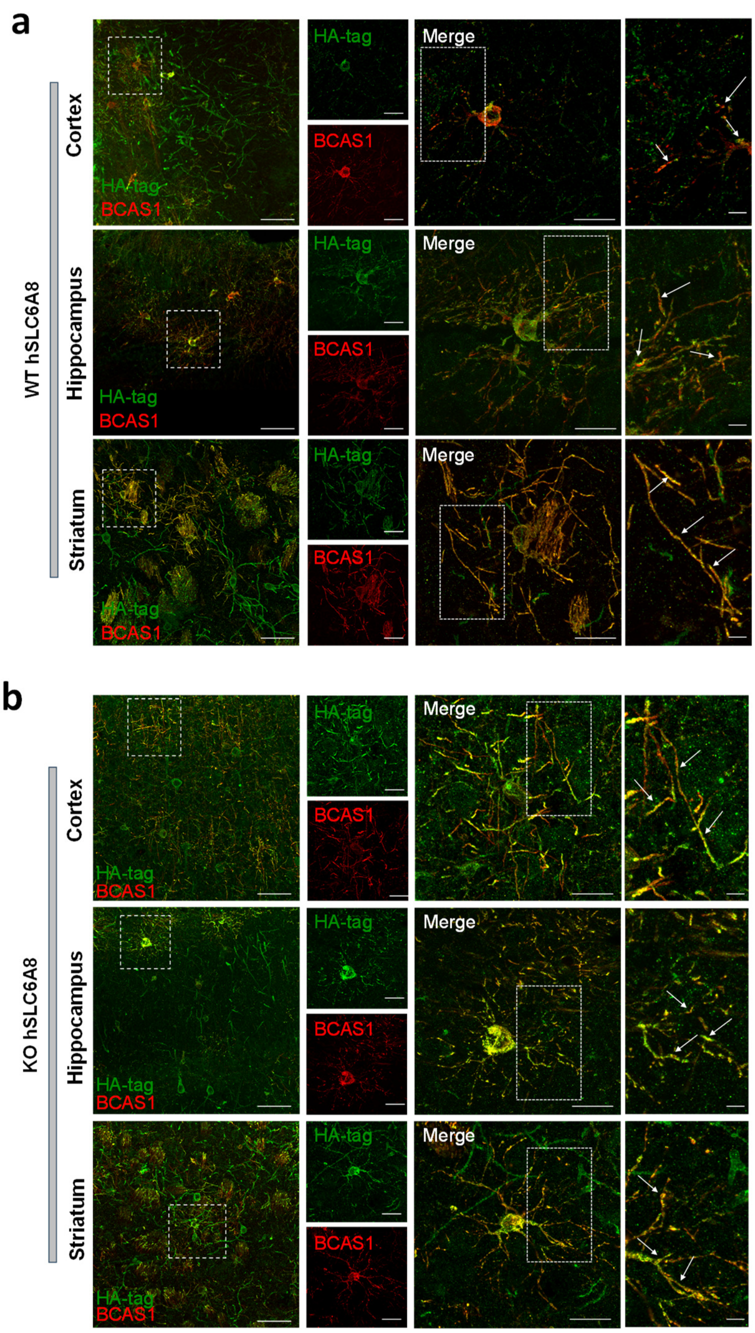

Supplementary Figure *7.* ***Exogenous CRT protein is expressed in oligodendrocytes.*** *Representative images at 20x and 63x magnification, and relative insets illustrating the colocalization of HA-tag staining (green signal) with oligodendrocytic marker BCAS1 (red signal) in the cortex, hippocampus and striatum of WT* ***(a)*** *and Slc6a8-KO* ***(b)*** *mice injected with AAV-hSLC6A8. In both (a) and (b), images in the leftmost columns were acquired at 20x magnification (scale bars are 50 µm). Additional panels were acquired at 63x magnification (scale bars are 20 µm, 50 µm and 5 µm from left to right, respectively).*

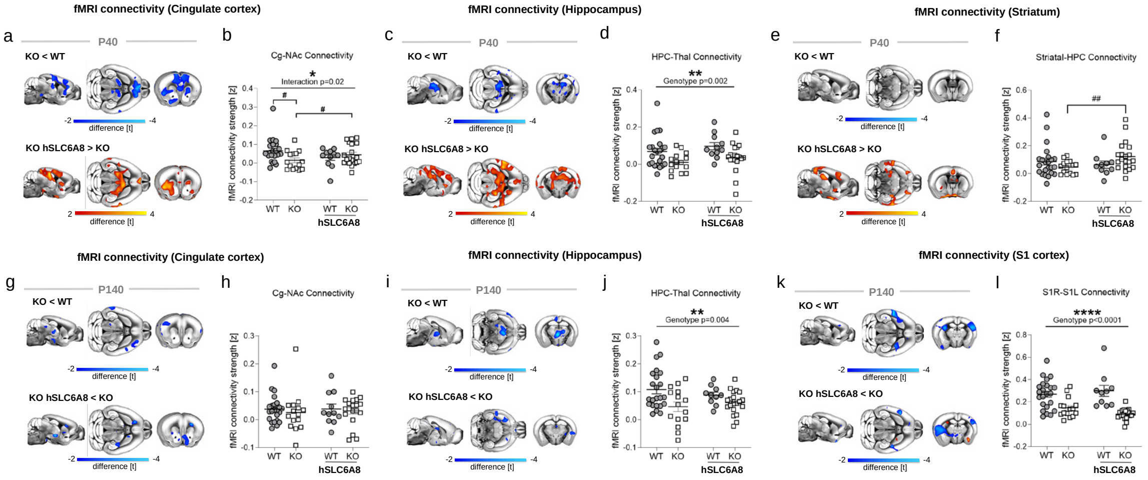

Supplementary Figure 8. AAV-hSLC6A8 treatment prevents onset of fMRI hypoconnectivity in Slc6a8-KO mice. *(**a-f**) Seed-correlation mapping highlighted ameliorated fronto-limbic hypoconnectivity in treated Slc6a8-KO mice at P40. Red/yellow coloring in bottom panels denotes increased connectivity produced by AAV- hSLC6A8 treatment (t-test, t> 2). Histograms illustrate quantification of connectivity strength between the probed seed and regions of interest (b, Cg-NAc: two-way ANOVA, genotype x treatment interaction F = 5.24, p = 0.025; t-test, # p < 0.05; d, HPC-Thal: two-way ANOVA, genotype factor F = 10.7, p = 0.0017, interaction, F = 0.01, p = 0.92; f, striatal-HPC: two-way ANOVA, interaction F = 3.94, p = 0.051; t-test, ## p < 0.01). (**g-l**) The same analysis at P140 revealed no effect of gene therapy (h, Cg-NAc: two-way ANOVA, interaction F = 0.03, p = 0.85; j, HPC-Thal: two-way ANOVA, genotype factor F = 8.77, p = 0.004, interaction, F = 1.33, p = 0.25; l, S1R-S1L: two-way ANOVA, genotype factor F = 36.5, p < 0.0001, interaction F = 2.60, p = 0.11). HPC, hippocampus; Ins, insula; Thal, thalamus; Cg, cingulate cortex; NAc, Nucleus Accumbens; S1R, right somatosensory cortex 1; S1L, left somatosensory cortex 1. All statistics are FWE cluster-corrected. FWE, family-wise error. For two-way ANOVA, * p< 0.05, **p< 0.01, ****p < 0.0001. Error bars indicate SEM and dots represent individual values.*

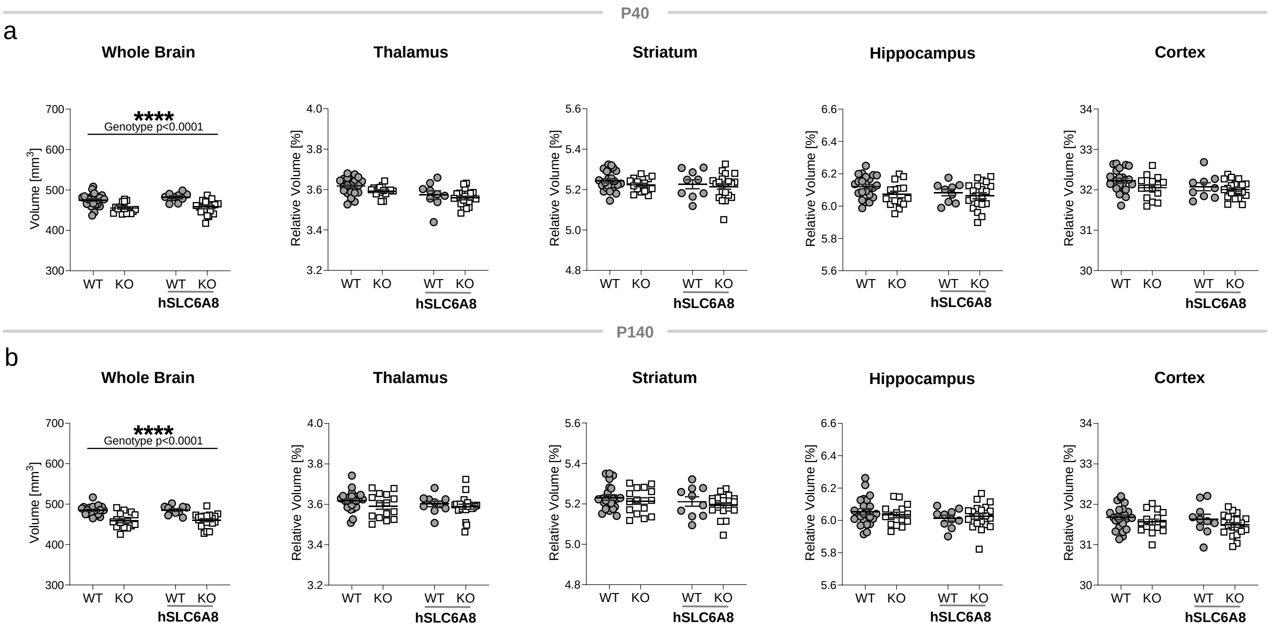

Supplementary Figure 9. Reduced whole brain volume in Slc6a8-KO mice. *(**a**) Whole and regional brain volume quantification in Slc6a8-KO and WT mice at P40 (two-way ANOVA; genotype $F = 30.96$, $p < 0.0001$; genotype x treatment interaction: $F = 0.32$, $p = 0.57$). Region-specific volumes at P40 (Thalamus, two-way ANOVA; genotype $F = 3.5$, $p = 0.066$, interaction $F = 0.17$, $p = 0.68$; Striatum, genotype factor $F = 1.41$, $p = 0.24$, interaction $F = 0.078$, $p = 0.78$; Hippocampus, genotype factor $F = 3.09$, $p = 0.08$, interaction $F = 0.59$, $p = 0.44$; Cortex, two-way ANOVA, genotype factor $F = 2.95$, $p = 0.09$, interaction $F = 0.51$, $p = 0.47$). (**b**) Whole and regional brain volume quantification in Slc6a8-KO and WT mice at P140 (two-way ANOVA; genotype factor $F = 44.5$, $p < 0.0001$; interaction $F = 0.0007$, $p = 0.98$). Region-specific volumes at P140 (Thalamus, genotype factor $F = 2.12$, $p = 0.15$, interaction $F = 0.18$, $p = 0.67$; Striatum, genotype factor $F = 1.18$, $p = 0.28$, interaction $F = 0.0009$, $p = 0.98$; Hippocampus, genotype factor $F = 0.04$, $p = 0.85$, interaction $F = 0.74$, $p = 0.39$; Cortex, two-way ANOVA, genotype factor $F = 2.89$, $p = 0.09$, interaction $F = 0.037$, $p = 0.85$). Plots report absolute volume in mm^3 and relative volume % normalized to whole brain volume, error bars indicate SEM; dots represent individual values; ****$p < 0.0001$.*

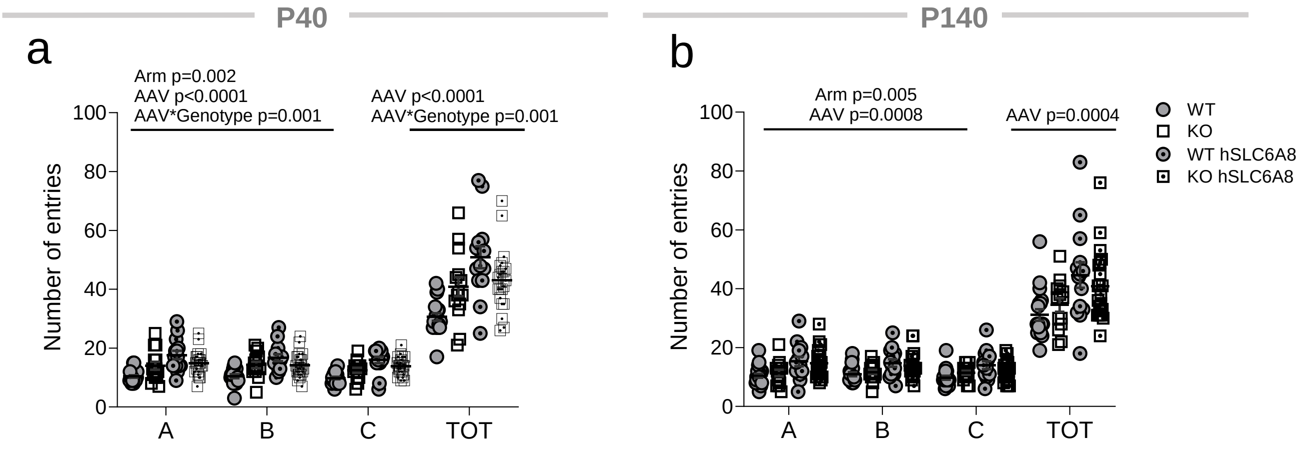

Supplementary Figure 10. Behavioral performance following treatment with AAV-hSLC6A8. *Histograms depict the mean number of entries in the single arms of the maze (A, B, C) and the total number of arm entries (TOT) for the different experimental groups at P40 (**a**) and P140 (**b**). An increased number of entries was found in mice injected with AAV-hSLC6A8 both at P40 (single arms: Three-way RM ANOVA, factor AAV $F = 18.8$, $p < 0.0001$; TOT: two-way ANOVA factor AAV $F= 20.05$, $p < 0.0001$) and P140 (single arms: Three-way RM ANOVA, factor AAV $F = 12.42$, $p = 0.0008$; TOT: two-way ANOVA factor AAV $F= 13.9$, $p = 0.0004$).*

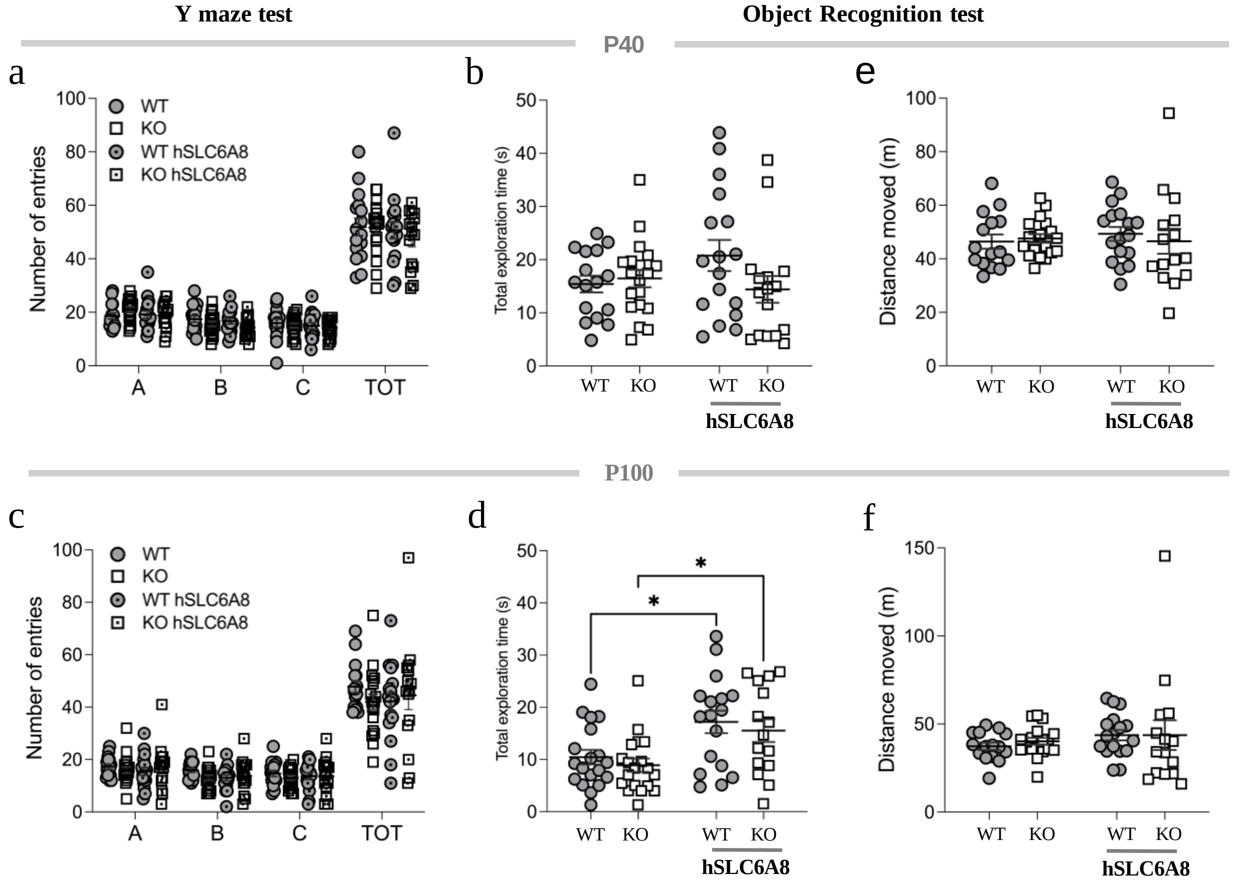

Supplementary Figure 11. Perinatal AAV-hSLC6A8 injection does not alter overall exploratory behavior in WT and Slc6a8-KO mice. *(**a**) Number of entries in the Y-maze task (three-way ANOVA, interaction F = 0.540, p = 0.655) and (**b**) total exploration time in the Object Recognition Test (ORT) in the four experimental groups at P40 (two-way ANOVA, interaction F = 2.76, p = 0.102). At P100, the number of Y-maze entries remained unaffected across experimental groups (**c**, three-way ANOVA, interaction F = 1.15, p = 0.330), whereas object exploration time significantly increased in both genotypes following AAV-hSLC6A8 administration (**d**, two-way ANOVA, treatment F = 14.73, p = 0.0003). (**e-f**) Total distance moved in the ORT arena at P40 (e, two-way ANOVA, interaction F = 0.44, p = 0.51) and P100 (f, two- way ANOVA, interaction F = 0.101, p = 0.752). Error bars indicate SEM, each dot represents a mouse. Tukey's multiple comparison test, *p < 0.05.*

Creatine Quantification

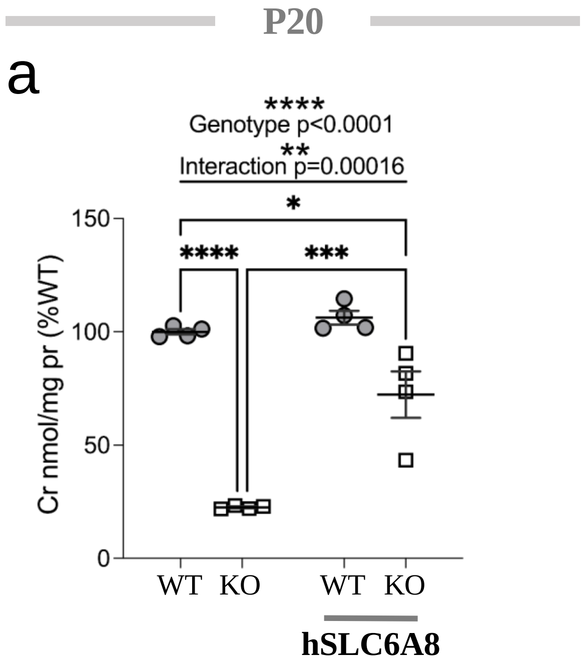

Supplementary Figure 12. Quantification of brain creatine levels at P20 *(two-way ANOVA, genotype factor F = 107.1 p < 0.0001, treatment F = 27.1, p = 0.0002, interaction F =16.3, p = 0.0016). Error bars indicate SEM and each dot represents a mouse. Tukey's multiple comparison test, *p < 0.05, ***p < 0.001, ****p < 0.0001.*

Table S1. Full sequence of pAAV-Jet-hSLC6A8

cttccgcttcctcgctcactgactcgctgcgctcggtcgttcggctgcggcgagcggtatcagctcactcaaaggcggtaatacggttatccacagaatcaggggataacgcaggaaagaacatgtgagcaaaaggccagcaaaaggccagg
aaccgtaaaaaggccgcgttgctggcgtttttccataggctccgcccccctgacgagcatcacaaaaatcgacgctcaagtcagaggtggcgaaacccgacaggactataaagataccaggcgtttccccctggaagctccctcgtgcgctc
tcctgttccgaccctgccgcttaccggatacctgtccgcctttctcccttcgggaagcgtggcgctttctcatagctcacgctgtaggtatctcagttcggtgtaggtcgttcgctccaagctgggctgtgtgcacgaaccccccgttcagcccg
accgctgcgccttatccggtaactatcgtcttgagtccaacccggtaagacacgacttatcgccactggcagcagccactggtaacaggattagcagagcgaggtatgtaggcggtgctacagagttcttgaagtggtggcctaactacggcta
cactagaagaacagtatttggtatctgcgctctgctgaagccagttaccttcggaaaaagagttggtagctcttgatccggcaaacaaaccaccgctggtagcggtggtttttttgtttgcaagcagcagattacgcgcagaaaaaaaggatctc
aagaagatcctttgatcttttctacggggtctgacgctcagtggaacgaaaactcacgttaagggattttggtcatgagattatcaaaaaggatcttcacctagatccttttaaattaaaaatgaagttttaaatcaatctaaagtatatatgagtaa
acttggtctgacagttaccaatgcttaatcagtgaggcacctatctcagcgatctgtctatttcgttcatccatagttgcctgactccccgtcgtgtagataactacgatacgggagggcttaccatctggccccagtgctgcaatgataccgcg
agacccacgctcaccggctccagatttatcagcaataaaccagccagccggaagggccgagcgcagaagtggtcctgcaactttatccgcctccatccagtctattaattgttgccgggaagctagagtaagtagttcgccagttaatagtttg
cgcaacgttgttgccattgctacaggcatcgtggtgtcacgctcgtcgtttggtatggcttcattcagctccggttcccaacgatcaaggcgagttacatgatcccccatgttgtgcaaaaaagcggttagctccttcggtcctccgatcgttgtc
agaagtaagttggccgcagtgttatcactcatggttatggcagcactgcataattctcttactgtcatgccatccgtaagatgcttttctgtgactggtgagtactcaaccaagtcattctgagaatagtgtatgcggcgaccgagttgctcttgcc
cggcgtcaatacgggataataccgcgccacatagcagaactttaaaagtgctcatcattggaaaacgttcttcggggcgaaaactctcaaggatcttaccgctgttgagatccagttcgatgtaacccactcgtgcacccaactgatcttcagc
atcttttactttcaccagcgtttctgggtgagcaaaaacaggaaggcaaaatgccgcaaaaaagggaataagggcgacacggaaatgttgaatactcatactcttcctttttcaatattattgaagcatttatcagggttattgtctcatgagcgg
atacatatttgaatgtatttagaaaaataaacaaataggggttccgcgcacatttccccgaaaagtgccacctaaattgtaagcgttaatattttgttaaaattcgcgttaaatttttgttaaatcagctcattttttaaccaataggccgaaatcgg
caaaatcccttataaatcaaaagaatagaccgagatagggttgagtgttgttccagtttggaacaagagtccactattaaagaacgtggactccaacgtcaaagggcgaaaaaccgtctatcagggcgatggcccactacgtgaaccatcacc
ctaatcaagttttttggggtcgaggtgccgtaaagcactaaatcggaaccctaaagggagcccccgatttagagcttgacggggaaagccggcgaacgtggcgagaaaggaagggaagaaagcgaaaggagcgggcgctagggcgctggca
agtgtagcggtcacgctgcgcgtaaccaccacacccgccgcgcttaatgcgccgctacagggcgcgtcccattcgccattcaggctgcgcaactgttgggaagggcgatcggtgcgggcctcttcgctattacgccagctgcgcgctcgctc
gctcactgaggccgcccgggcaaagcccgggcgtcgggcgacctttggtcgcccggcctcagtgagcgagcgagcgcgcagagagggagtggccaactccatcactaggggttccttgtagttaatgattaacccgccatgctacttatcta
cgtagccatgctctaggaagagtaccattgacgtcaataatgacgtatgttcccatagtaacgccaatagggactttccattgacgtcaatgggtggagtatttacggtaaactgcccacttggcagtacatcaagtgtatcatatggggcggag
ttagggcggagccaatcagcgtgcgccgttccgaaagttgccttttatggctgggcggagaatgggcggtgaacgccgatgattatataaggacgcgccgggtgtggcacagctagttccgtcgcagccgggatttgggtcgcggttcttgtttg
tggggttcggcttctggcgtgtgaccggcggctctagagccaccatggcgaagaagagcgccgagaacggcatctatagcgtgtccggcgacgagaagaagggcccccctcatcgcgcccgggcccgacggggccccggccaagggcgacg
gccccgtgggcctggggacacccggcggccgcctggccgtgccgccgcgcgagacctggacgcgccagatggacttcatcatgtcgtgcgtgggcttcgccgtgggcttgggcaacgtgtggcgcttcccctacctgtgctacaagaacgg
cggaggtgtgttccttattccctacgtcctgatcgccctggttggaggaatccccattttcttcttagagatctcgctgggccagttcatgaaggccggcagcatcaatgtctggaacatctgtcccctgttcaaaggcctgggctacgcctccat
ggtgatcgtcttctactgcaacacctactacatcatggtgctggcctggggcttctattacctggtcaagtcctttaccaccacgctgccctgggccacatgtggccacaTctggaacactcccgactgcgtggagatcttccgccatgaagac
tgtgccaatgccagcctggccaacctcacctgtgaccagcttgctgaccgccggtcccctgtcatcgagttctgggagaacaaagtcttgaggctgtctgggggactggaggtgccaggggccctcaactgggaggtgaccctttgtctgctg
gcctgctgggtgctggtctacttctgtgtctggaagggggtcaaatccacgggaaagatcgtgtacttcactgctacattcccctacgtggtcctggtcgtgctgctggtgcgtggagtgctgctgcctggcgccctggatggcatcatttactat
ctcaagcctgactggtcaaagctggggtcccctcaggtgtggatagatgcggggacccagattttcttttcttacgccattggcctgggggccctcacagccctgggcagctacaaccgcttcaacaacaactgctacaaggacgccatcatc
ctggctctcatcaacagtgggaccagcttctttgctggcttcgtggtcttctccatcctgggcttcatggctgcagagcagggcgtgcacatctccaaggtggcagagtcagggccgggcctggccttcatcgcctacccgcgggctgtcacgc
tgatgccagtggccccactctgggctgccctgttcttcttcatgctgttgctgcttggtctcgacagccagtttgtaggtgtggagggcttcatcaccggcctcctcgacctcctcccggcctcctactacttccgtttccaaagggagatctctg
tggccctctgttgtgccctctgctttgtcatcgatctctccatggtgactgatggcgggatgtacgtcttccagctgtttgactactactcggccagcggcaccaccctgctctggcaggccttttgggagtgcgtggtggtggcctgggtgtacg
gagctgaccgcttcatggacgacattgcctgtatgatcgggtaccgaccttgcccctggatgaaatggtgctggtccttcttcaccccgctggtctgcatgggcatcttcatcttcaacgttgtgtactacgagccgctggtctacaacaacacc
tacgtgtacccgtggtggggtgaggccatgggctgggccttcgccctgtcctccatgctgtgcgtgccgctgcacctcctgggctgcctcctcagggccaagggcaccatggctgagcgctggcagcacctgacccagcccatctggggcct
ccaccacttggagtaccgagctcaggacgcagatgtcaggggcctgaccaccctgaccccagtgtccgagagcagcaaggtcgtcgtggtggagagtgtcatgtacccatacgatgttccagattacgcatgaggatccgagctcggtacca
agcttatcgataatcaacctctggattacaaaatttgtgaaagattgactggtattcttaactatgttgctccttttacgctatgtggatacgctgctttaatgcctttgtatcatgctattgcttcccgtatggctttcattttctcctccttgtataaa
tcctggttgctgtctctttatgaggagttgtggcccgttgtcaggcaacgtggcgtggtgtgcactgtgtttgctgacgcaacccccactggttggggcattgccaccacctgtcagctcctttccgggactttcgctttccccctccctattgcc
acggcggaactcatcgccgcctgccttgcccgctgctggacaggggctcggctgttgggcactgacaattccgtggtgttgtcggggaaatcatcgtcctttccttggctgctcgcctgtgttgccacctggattctgcgcgggacgtccttctg
ctacgtcccttcggccctcaatccagcggaccttccttcccgcggcctgctgccggctctgcggcctcttccgcgtcttcgccttcgccctcagacgagtcggatctccctttgggccgcctccccgcatcgataccgtcgacccgggcggcc
gcttcgagcagacatgataagatacattgatgagtttggacaaaccacaactagaatgcagtgaaaaaaatgctttatttgtgaaatttgtgatgctattgctttatttgtaaccattataagctgcaataaacaagttaacaacaacaattgcatt
cattttatgtttcaggttcagggggagatgtgggaggttttttaaagcaagtaaaacctctacaaatgtggtaaaatcgataaggatcttcctagagcatggctacgtagataagtagcatggcgggttaatcattaactacaaggaacccctagt
gatggagttggccactccctctctgcgcgctcgctcgctcactgaggccgggcgaccaaaggtcgcccgacgcccgggctttgcccgggcggcctcagtgagcgagcgagcgcgcagctgcattaatgaatcggccaacgcgcggggagag
gcggtttgcgtattgggcgct

Table S2. Complete list of brain areas (and abbreviations) used for NBS. 85 anatomically parcellated regions as in Coletta et al., 2020. ISO: isocortex, OLF: olfactory region, HPF: hippocampal formation, CTXsp: cortical subplate, STR:striatum, THAL: thalamus, HY: hypothalamus, MID: midbrain

1	MOp	Primary motor area	ISO	43	PVR	Periventricular region	HY
2	MOs	Secondary motor area	ISO	44	MEZ	Hypothalamic medial zone	HY
3	Ssp	Primary somatosensory area	ISO	45	LZ	Hypothalamic lateral zone	HY
4	SSs	Supplemental somatosensory area	ISO	46	MBsen	Midbrain, sensory related	MID
5	GU	Gustatory areas	ISO	47	MBmot	Midbrain, motor related	MID
6	VISC	Visceral area	ISO	48	MBsta	Midbrain, behavioral state related	MID
7	AUD	Auditory area	ISO	49	MOp	Primary motor area	ISO
8	VIS	Visual area	ISO	50	MOs	Secondary motor area	ISO
9	ACAd	Anterior cingulate area, dorsal part	ISO	51	Ssp	Primary somatosensory area	ISO
10	ACAv	Anterior cingulate area, ventral part	ISO	52	SSs	Supplemental somatosensory area	ISO
11	PL	Prelimbic area	ISO	53	GU	Gustatory areas	ISO
12	ILA	Infralimbic area	ISO	54	VISC	Visceral area	ISO
13	ORB	Orbital area	ISO	55	AUD	Auditory area	ISO
14	AId	Agranular insular area, dorsal part	ISO	56	VIS	Visual area	ISO
15	AIp	Agranular insular area, posterior part	ISO	57	AId	Agranular insular area, dorsal part	ISO
16	AIv	Agranular insular area, ventral part	ISO	58	AIp	Agranular insular area, posterior part	ISO
17	RSPagl	Retrosplenial area, lateral agranular part	ISO	59	AIv	Agranular insular area, ventral part	ISO
18	RSPd	Retrosplenial area, dorsal part	ISO	60	PTLp	Posterior parietal association areas	ISO
19	RSPv	Retrosplenial area, ventral part	ISO	61	TEa	Temporal association areas	ISO
20	PTLp	Posterior parietal association areas	ISO	62	PERI	Perirhinal area	ISO
21	TEa	Temporal association areas	ISO	63	ECT	Ectorhinal area	ISO
22	PERI	Perirhinal area	ISO	64	PIR	Piriform area	OLF
23	ECT	Ectorhinal area	ISO	65	OLFnuc	olfactory_nuclei	OLF
24	PIR	Piriform area	OLF	66	CA	Ammon's horn	HPF
25	OLFnuc	olfactory_nuclei	OLF	67	DG	Dentate gyrus	HPF
26	CA	Ammon's horn	HPF	68	ENT	Entorhinal area	HPF
27	DG	Dentate gyrus	HPF	69	RHP	Retro_hippocampal	HPF
28	ENT	Entorhinal area	HPF	70	EndCla	EndopiriformNucleus_Claustrum	CTXsp
29	RHP	Retro_hippocampal	HPF	71	LA	Lateral amygdalar nucleus	CTXsp
30	EndCla	EndopiriformNucleus_Claustrum	CTXsp	72	BLA	Basolateral amygdalar nucleus	CTXsp
31	LA	Lateral amygdalar nucleus	CTXsp	73	BMA	Basomedial amygdalar nucleus	CTXsp
32	BLA	Basolateral amygdalar nucleus	CTXsp	74	PA	Posterior amygdalar nucleus	CTXsp
33	BMA	Basomedial amygdalar nucleus	CTXsp	75	STRd	Striatum dorsal region	STR
34	PA	Posterior amygdalar nucleus	CTXsp	76	STRv	Striatum ventral region	STR
35	STRd	Striatum dorsal region	STR	77	LSX	Lateral septal complex	STR
36	STRv	Striatum ventral region	STR	78	sAMY	Striatum-like amygdalar nuclei	STR
37	LSX	Lateral septal complex	STR	79	PAL	Pallidum	PAL
38	sAMY	Striatum-like amygdalar nuclei	STR	80	DORsm	Thalamus, sensory-motor cortex related	THAL
39	PAL	Pallidum	PAL	81	DORpm	Thalamus, polymodal association cortex related	THAL
40	DORsm	Thalamus, sensory-motor cortex related	THAL	82	LZ	Hypothalamic lateral zone	HY
41	DORpm	Thalamus, polymodal association cortex related	THAL	83	MBsen	Midbrain, sensory related	MID
42	PVZ	Periventricular zone	HY	84	MBmot	Midbrain, motor related	MID
				85	MBsta	Midbrain, behavioral state related	MID
